# Troubles de la ménopause: enquête sur les connaissances, attitudes et pratiques du personnel des structures sanitaires de Dakar

**DOI:** 10.11604/pamj.2014.18.257.3156

**Published:** 2014-07-27

**Authors:** Abdoul Aziz Diouf, Marie Edouard Faye-Diémé, Mamour Guèye, Tatiana Gisèle Sandjon, Magatte Mbaye, Jean Charles Moreau, Alassane Diouf

**Affiliations:** 1 UCAD, Clinique Gynécologique et Obstétricale, Dakar, Sénégal

**Keywords:** Ménopause, enquête CAP, Sénégal, menopause, KAP survey, Senegal

## Abstract

**Introduction:**

Evaluer le niveau de connaissance du personnel de santé des structures sanitaires de la région de Dakar, l'attitude et les pratiques face aux troubles de la ménopause.

**Méthodes:**

Enquête prospective auprès de 135 prestataires avec un questionnaire anonyme divisé en quatre rubriques: les caractéristiques socioprofessionnelles, les connaissances générales sur la ménopause, l'attitude et la pratique du prestataire par rapport à la ménopause troublée.

**Résultats:**

Les prestataires de santé inclus dans l’étude étaient répartis comme suit: 11 gynécologues, 37 médecins en spécialisation gynécologique, 34 médecins généralistes et 53 sages-femmes d’état. L’âge moyen était de 35 ans. Le sexe féminin représentait 64% du groupe. Vingt et un pourcent des prestataires exerçaient depuis plus de 10 ans. De manière globale, les connaissances générales sur la ménopause étaient satisfaisantes du moment où la majeure partie des prestataires était en mesure de poser le diagnostic et de prévoir les conséquences du déficit hormonal. La majorité des prestataires (62%) était favorable au traitement de la ménopause troublée. Cependant, nous notons une certaine insuffisance dans le traitement de la ménopause, aussi bien dans le cadre de la thérapie hormonale que dans l'utilisation des moyens alternatifs.

**Conclusion:**

Le nombre de femmes ménopausées augmente progressivement, et il convient de réunir toutes les stratégies de mise à niveau afin de faire face au défit de l'amélioration de la qualité de vie de cette catégorie de la population.

## Introduction

Au Sénégal, on estime que 12.6% de la population féminine sont ménopausées [1]. Ce nombre va évoluer d'année en année avec l'augmentation progressive de l'espérance de vie des femmes. Si les remaniements physiologiques engendrés par la ménopause étaient acceptés autrefois comme une fatalité dont les femmes ne parlaient guère, de nos jours, les plaintes sont clairement exprimées. A l'heure actuelle, face à l'explosion de sources médiatiques véhiculant autant d'informations sérieuses que d'articles erronés, le personnel de santé est il apte à répondre aux interrogations des patientes ménopausées? Apte à conseiller chaque femme et à envisager avec elle les différentes options thérapeutiques disponibles? Telles étaient les questions auprès d'un échantillon du personnel de santé de quelques structures sanitaires de Dakar. Les objectifs étaient d'estimer leur niveau de connaissance concernant les troubles de la ménopause, de déterminer leurs attitudes et pratiques face aux troubles de la ménopause et d’évaluer leurs besoins de formation sur le sujet.

## Méthodes

Il s'agissait d'une enquête prospective sur les connaissances, attitudes et pratique des prestataires de santé exerçant dans les secteurs public et privé de Dakar pendant la période de Mars à Juillet 2011. Etaient inclus de l’étude: les gynécologues en exercice dans la région de Dakar, les médecins en spécialisation gynécologique, les médecins généralistes et les sages-femmes d’état de la Région Médicale de Dakar. Tout personnel de santé obéissant aux critères d'inclusion mais refusant de participer ou absent de son poste au moment de l'enquête était exclu de l’étude.

La fiche d'enquête était anonyme, avec des questions ouvertes, fermées ou à choix multiples. Le questionnaire était divisé en cinq volets: les caractéristiques socioprofessionnelles, les connaissances générales sur la ménopause, l'attitude et la pratique du prestataire par rapport à la ménopause troublée et l’évaluation des besoins de formation sur le sujet. La fiche d'enquête a été testée au préalable sur quelques professionnels pour évaluer la compréhension des questions, la qualité des réponses et estimer le temps moyen de réponse. Dans chaque structure, après l'autorisation du médecin chef, les questionnaires étaient remis à toute personne répondant aux critères d'inclusion. Après une explication succincte concernant l'enquête, le personnel de santé remplissait lui-même le questionnaire. A cause du volume de travail, d'un calendrier particulièrement rempli pour certains ou même de la léthargie et du désintérêt pour d'autres, la collecte des résultats s’était très souvent effectuée en plusieurs étapes. Dans certains cas, des compléments d'informations étaient sollicités. Ainsi, 135 questionnaires étaient dûment remplis. Les données étaient saisies et analysées avec le logiciel SPSS.

## Résultats

### Caractéristiques socioprofessionnelles

**Figure 1:**
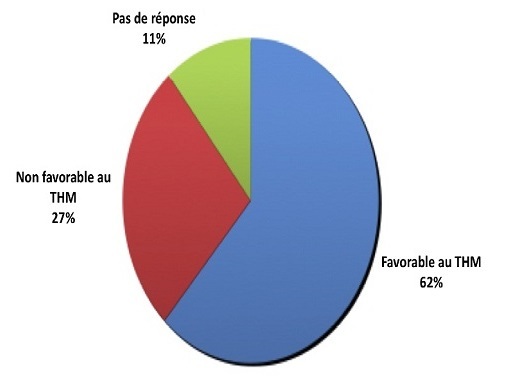
Répartition de la population d’étude suivant leur point de vue sur le THM et son indication lors des bouffées de chaleur

Les prestataires de santé inclus dans l’étude au nombre de 135 étaient répartis comme suit: 11 gynécologues, 37 médecins en spécialisation gynécologique, 34 médecins généralistes et 53 sages-femmes d’état (Tableau I). Ils étaient 49% à exercer dans les hôpitaux publics, 41% dans les centres de santé, 6% dans des cliniques privées, et juste 4% dans les postes de santé. La tranche d’âge des 30-39 ans était la plus représentée suivie de celle des 20-29 ans. Les femmes constituaient la majorité des personnes interrogées (64%); ce qui est aisément compréhensible vu que les sages-femmes étaient l'une des catégories professionnelles ciblées. Aussi, 13% des femmes avaient plus de 50 ans, donc très concernées par la ménopause et ses éventuels troubles. De la population d’étude, 21.5% exerçait depuis plus de 10 ans (Figure 1). Cette catégorie du personnel était déjà en exercice pendant cette dernière décennie qui a connu la grande polémique et le rebond d'intérêt sur la ménopause et son traitement.

**Tableau 1:** Caractéristiques socio-profesionnelles des prestataires

	Nombre	Pourcentage
**Catégories professionnelles**		
**Gynécologues**	11	8
**Médecins généralistes**	34	25
**Médecins en spécialisation**	37	28
**Sages-femmes**	53	39
**Sexe**		
Féminin	86	64
Masculin	47	36
**Age**		
20-29 ans	36	27
30-39 ans	69	51
40-49 ans	18	13
≥ 50 ans	12	9
**Années d'expérience**		
< 10 ans	107	78.5
≥ 10 ans	28	21.5
**Type de structure**		
Hôpitaux publics	66	49
Centres de santé	55	41
Privés	8	6
Postes de santé	6	4

### Connaissance de la menopause

Concernant la définition de la ménopause, 52.6% des prestataires avaient trouvé la bonne réponse; il s'agissait essentiellement des médecins en spécialisation (50%) et des gynécologues (50%). Par contre, 21.5% de la population d’étude assimilaient la ménopause à une aménorrhée de plus d'un an quel que soit l’âge de la femme. Deux sages-femmes et 2 médecins généralistes affirmaient ne pas connaître la réponse.

S'agissant des signes évocateurs de la ménopause, les bouffées de chaleur (97%) et l'aménorrhée secondaire de plus d'un an (81.5%) étaient les plus cités par les agents de santé, suivis des modifications gynécologiques (43%) telles que les irrégularités du cycle menstruel, la sécheresse et l'atrophie vaginale. Les troubles neuropsychiques et les troubles de l'humeur étaient cités par 30% des prestataires. De l'ensemble des répondants, 2.2% estimaient que l’âge de 50 ans serait un signe évocateur de la ménopause ainsi que l'absence de fécondation (0.7%). Quand aux signes biologiques de la ménopause, 48% de la population d’étude n'avaient pas d'idée sur les examens à proposer.

A la question de savoir les conséquences à long terme de la ménopause, l'ostéoporose ainsi que les fractures pathologiques (58.6%) étaient les anomalies les plus évoquées par le personnel de santé suivies des maladies cardio-vasculaires (29.6%), et des troubles neuropsychiques (11.9%). Si quelques praticiens avaient désigné des troubles oculaires (0.7%) et les métrorragies post-ménopausiques (0.7%) comme conséquences imputables à la ménopause, 25.9% de la population d’étude avaient avoué n'avoir aucune idée. Il en ressort que 78.5% des praticiens n'avaient jamais entendu parler des études scientifiques portant sur le traitement de la ménopause et leur impact sur la prise en charge des patientes. Les mieux informés (n = 24) étaient ceux qui exerçaient depuis moins de 9 ans. Les gynécologues en exercice (72.7%) avaient une meilleure connaissance de l’évaluation du THM et des études s'y rattachant, suivis des médecins en spécialisation (21.6%) et des sages femmes (17%). Les médecins généralistes (11.8%) étaient les moins informés. Des 29 prestataires ayant affirmé être à jour sur les évaluations du THM et les études associées, seuls 23% avaient mentionné l’étude américaine Women Health Initiative (WHI).

### Attitudes devant une ménopause trouble

Une grande proportion du personnel de santé (56.3%) recevait rarement des patientes pour des troubles de la ménopause lors de leurs consultations, c′est-à-dire au plus, une fois par mois. Quelques 27.4% les recevaient occasionnellement (environ une fois toutes les deux semaines), essentiellement les gynécologues.

Par contre, 5 médecins généralistes, 5 sages-femmes et un médecin en spécialisation n'avaient jamais reçu de patientes venant consulter pour des troubles de la ménopause. Parmi les praticiens qui en recevaient, 91% des gynécologues les prenaient en charge eux mêmes, 27% des médecins en spécialisation et 70.6% des généralistes référaient les patientes ménopausées.

Parmi les prestataires interrogés, 62% estimaient que le THM se justifie devant les troubles climatériques, spécialement les bouffées de chaleur contre 27% qui n'en voyaient pas l'utilité.

De ces 62% ayant répondu par l'affirmative, 45.2% estimaient que le THM peut être un facteur de risque de cancer.

Pour ce qui est de la mammographie, juste 52.6% en demandaient systématiquement. Par contre, ils étaient 75.6% à proposer un FCV (86.5% des médecins en spécialisation et 100% des gynécologues). Les plus modestes en demande d'examens complémentaires étaient les médecins généralistes avec 75% d'entre eux qui affirmaient ne pas faire de suivi de femmes ménopausées.

### Questions sur la pratique

Une proportion de 45% des enquêtés admettaient avoir eu à prescrire un traitement contre les bouffées de chaleur. De ce nombre, les sages femmes semblaient majoritaires du fait de leur effectif élevé quand on considère l'ensemble des prestataires. L'habitude la plus commune est de prescrire pour une durée de 1 à 2 ans. Cinq% affirmaient avoir déjà prescrit le THM sur une durée de plus de 7 ans. Cependant 54.1% (soit plus de la moitié de l’échantillon) avaient avoué ne pas connaitre les contre indications du THM. En pratique, 61.5% des praticiens prescrivaient des phytoestrogènes comme traitement alternatif tandis que 24.4% ne prescrivaient aucune thérapie (Tableau II). De notre population d’étude, 43% affirmaient n'avoir pas reçu d'enseignement portant sur la ménopause dans le cadre de leur formation (37,8% des médecins en spécialisation et 50% des médecins généralistes). Ils étaient 6.7% à trouver la formation portant sur la ménopause faite pendant leurs cursus universitaire, suffisante. Une proportion de 61.4% la jugeait insuffisante. Ainsi, 94.1% du personnel étaient disposés à recevoir une formation complémentaire sur le sujet.

**Tableau 2:** Les différents traitements institués par les prestataires face aux bouffées de chaleur

	Sages-femmes		Médecins		Total	
	Effectif	%	Effectif	%	Effectif	%
THM	0	0	5	3,7	5	3.7
Phytoestrogènes	29	21.5	54	40	83	61.5
Tibolone	0	0	1	0.7	1	0.7
Acupuncture	1	0.7	4	3	5	3.7
SERM	0	0	3	2,2	3	2.2
Autres	3	2.2	14	10.3	17	12.6
Ne prescrivent pas	21	15.5	12	8.9	33	24.4

## Discussion

Les catégories socioprofessionnelles que nous avons choisies dans notre étude sont assez représentatives de notre système sanitaire: le nombre de médecins est insuffisant et très mal réparti (0.059 pour 1000 habitants) [2] tandis que les sages-femmes sont beaucoup plus en proximité avec la population. Ces dernières sont des professionnelles que les patientes consultent en première ligne quels que soient leur âge et leurs problèmes de santé. Bien qu'elle ne soit pas stipulée clairement parmi les compétences qu'on leurs a reconnues et déléguées, rien ne nous permet d‘écarter la prise en charge de la ménopause de leurs activités courantes. De ce fait, les sages-femmes gardent une place primordiale dans la prise en charge initiale de la ménopause troublée. Il s'agit vraisemblablement d'une situation particulière qui ne peut être superposée à celle des pays développés où les sages-femmes ont leur domaine d'exercice clairement défini. Cependant, comme nous l'avons constaté dans notre étude, leur connaissance sur la ménopause était généralement insuffisante pour assurer une prise en charge correcte des formes troublées. Si 81.9% des prestataires affirmaient avoir reçu en consultation une patiente présentant une ménopause troublée, 77% n'avaient jamais entendu parler de l’étude WHI [3]. Pourtant, cette étude a marqué au plus haut point l'histoire du traitement hormonal de la ménopause. Ses résultats sur le risque de survenue du cancer mammaire et de maladies thrombo-emboliques avaient créé une véritable discorde au sein de la communauté des gynécologues européens et américains, semant le trouble chez les patientes concernées [4, 5]. Il s'en était suivi plusieurs enquêtes pour apprécier le changement de pratique des praticiens après ces accablants résultats. Blümmel et al. [6] avaient étudié les effets de la WHI sur les médecins exerçant sur le territoire américain. Selon leurs résultats, 97.2% des 283 médecins interrogés prenaient en compte, pour leur prescription de THM, les conclusions de la WHI, et 64.7% d'entre eux avaient modifié leur approche thérapeutique (réduction des doses et de la durée du THM entre autres). Quant à l’étude de Ettinger et al. [7], 56% des 670 femmes de 50 à 69 ans interrogées avaient arrêté le THM au cours des six à huit mois suivant la publication princeps de l’étude WHI. L'expérience subsaharienne sur le sujet est difficile à apprécier par manque d’études comparatives. Une étude épidémiologique que nous avions réalisée en 2008 avait d'ailleurs montré que seulement 2% des femmes sénégalaises ménopausées étaient sous THM. Le faible taux de prescription du THM contraste cependant avec l'utilisation à la limite religieuse des phytoestrogènes comme traitement alternatif qui concernait 61.5% des praticiens. En effet, l'usage des phytoestrogènes dans la prise en charge des symptômes climatériques est bien adapté à nos réalités. Les contre-indications sont moins nombreuses, le suivi et l'observance moins contraignants, la prescription plus facile. De ce fait, ils sont facilement maîtrisés par personnel paramédical qui est notamment le premier répondant des populations dans la gestion des problèmes de santé de la reproduction. Toutefois, il convient de rappeler que les phytoestrogènes ne peuvent nullement remplacer le THM, et à cet effet, ils doivent être utilisés comme des compléments alimentaires.

Le constat global sur la prise en charge médicale de la ménopause dans nos régions est marqué par une profonde insuffisance tant sur le plan de la formation des prestataires, que sur le suivi des femmes ménopausées. Il devient nécessaire d’élaborer des protocoles de prise en charge de la ménopause et de mettre en place des sociétés savantes de recherche sur la ménopause à l'image de l'Association française d’étude sur la ménopause (AFEM) ou celle marocaine (AMEM). Ces initiatives pourraient permettre de relever le niveau de connaissance et de pratique en matière de suivi des femmes ménopausées. C'est dans ce sens que 94,1% des prestataires étaient disposés à recevoir une formation sur ce sujet.

## Conclusion

L'enquête sur les connaissances, attitudes et pratiques de la ménopause dans nos régions nous révèle un véritable déficit qu'il faudra corriger par un renforcement des compétences des prestataires dans ce domaine. Le nombre de femmes ménopausées augmente progressivement, et il convient de réunir toutes les stratégies de mise à niveau afin de faire face au défit de l'amélioration de la qualité de vie de cette catégorie de la population.

## References

[1] Cisse CT, Diouf AA, Dieng T, Gueye Dieye A, Moreau JC (2008). Ménopause en milieu africain: épidémiologie, vécu et prise en charge à Dakar.

[2] Agence Nationale de la Statistique et de la Démographie Enquête Démographique et de Santé à Indicateurs Multiples Sénégal 2010-2011.

[3] Writing Group for the Women's Health Initiative Investigators (2002). Risks and benefits of estrogen plus progestin in healthy postmenopausal women: principal results from the Women's Health Initiative randomized controlled trial. JAMA.

[4] Jamin C, Raccah-Tebeka B, Chevallier T, Micheletti M.-C (2006). Impact de l’étude WHI sur le comportement des femmes médecins vis-à-vis de la ménopause. Gynecol Obstet Fertil..

[5] Jamin C, Raccah-Tebeka B (2003). Enquête Femme: la Femme médecin et la ménopause. La féminité à 50 ans.

[6] Ettinger B, Grady D, Tosteson AN, Pressman A, Macer JL (2003). Effect of the women's health initiative on women's decisions to discontinue postmenopausal hormone therapy. Obstet Gynecol..

[7] Blümmel JE, Castelo-Branco C, Chedraui PA, Binfa L, Dowlani B, Gomez MS (2004). Patients’ and clinicians’ attitudes after the women's health initiative study. Menopause.

